# Large esophageal schwannoma: En-bloc resection with primary closure by esophagoplasty^⋆^

**DOI:** 10.1016/j.ijscr.2019.07.038

**Published:** 2019-07-19

**Authors:** Jad A. Degheili, Pierre Sfeir, Ibrahim Khalifeh, Ali H. Hallal

**Affiliations:** a Division of General Surgery, Department of Surgery, American University of Beirut – Medical Center, Riad El-Solh 1107 2020, Beirut, Lebanon; b Department of Pathology and Laboratory Medicine, American University of Beirut – Medical Center, Riad El-Solh 1107 2020, Beirut, Lebanon

**Keywords:** Esophagus, Schwannoma, Submucosal tumor, Gastrointestinal stromal tumor, Esophagotomy, Esophagoplasty

## Abstract

**Background:**

Gastrointestinal schwannomas are submucosal tumors accounting for 2–7% of mesenchymal gastro-intestinal neoplasms; the stomach being the most common site. Esophageal schwannomas are more frequent in women, and are usually located in the upper to mid portion. Dysphagia is the main presenting symptom. A definitive diagnosis requires confirmation by histopathological and immunohistochemical studies.

**Case Presentation:**

A 50-year-old healthy lady, presented with gradual increasing onset of dyspnea, with minimal dysphagia to solid food, over a period of several years. Enhanced CT scan of the chest revealed a well-defined soft tissue mass arising from the proximal third of the esophagus, measuring 7.8 × 5.4 x 10.5 cm. Esophagogastric endoscopy with ultrasonography showed an elevated, smooth surface lesion, arising from the submucosal layer of the esophagus, with a hypervascular mucosa. Enucleation of this large tumor, with preservation of the overlying mucosa, was difficult to accomplish due to its large size.

Making use of a dilated proximal esophageal segment, total en-bloc excision of the mass rendered a 15 cm esophagotomy gap, which was easily closed, in two layers, without affecting the overall caliber thus achieving a good esophagoplasty result.

Histologically, abundance of spindle-shaped cells with positive S-100 proteins, confirmed the diagnosis of esophageal schwannoma.

**Conclusion:**

Variations in mesenchymal gastrointestinal tumors is vast, rendering diagnosis by radiology alone difficult. As such, characteristic histologic and immunostaining features are cornerstones in precise diagnosis of esophageal schwannomas. Despite being rare in incidence, symptomatic esophageal schwannoma lesions can be excised entirely, with low rate of recurrence and favorable overall outcomes.

## Highlights

• Gastrointestinal schwannomas are submucosal tumors accounting for 2–7% of mesenchymal gastro-intestinal neoplasms.
• Esophageal schwannomas are more frequent in women, and are usually located in the upper to mid portion.
• Symptomatic esophageal schwannomas can be excised entirely, with low rate of recurrence and favorable overall outcomes.
• Large esophageal schwannomas resulting in dilated proximal segment, esophagoplasty should be considered for defect closure.

## 1 Introduction

Esophageal tumors are seldom benign, and around 1% are detected clinically and radiologically [1]. The most frequently encountered are leiomyomas [1], and represent around 80% of benign esophageal tumors [2]. Esophageal schwannomas, on the other hand, are exceedingly rare, and constitute less than 2% of all esophageal tumors [3]. Esophageal schwannomas are initially described by Chaterlin and Fissore in 1967 [4]. Schwannomas are neurogenic tumors present within the mediastinum [1]. While having odynophagia, dysphagia, and/or shortness of breath as common presenting symptoms [1], esophageal schwannomas may be incidentally detected [5]. We hereby present the first reported case of a huge benign schwannoma located within the thoracic portion of the esophagus, for which complete excision of the mass and closure through an esophagoplasty was successfully performed. The present work has been reported in accordance with the SCARE criteria [6].

## 2 Case presentation

A 50-year-old healthy lady, case of bilateral partial mastectomy for high grade ductal carcinoma in situ, presented in 2014 with progressive exertional dyspnea, with minimal dysphagia to solid food, over a period of several years.

**Fig. 1:**
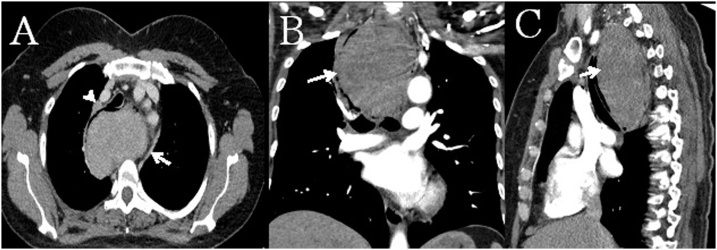
Computed Tomography (CT) Angiography of the chest with arterial and venous phase revealing a well-defined soft tissue mass (*arrow*) within the lumen of the esophagus at its proximal third measuring around 7.8 × 5.4 x 10.5 cm in its maximal transverse, AP, and craniocaudal dimensions respectively. It shows a smooth contour with mild enhancement on the arterial phase, which increases on the venous phase images. There is no extraluminal extension. A 7 mm lymph node (*arrowhead*), adjacent to the esophagus in the upper mediastinum, is also noted. (A: axial; B: coronal; C: sagittal cuts).

**Fig. 2:**
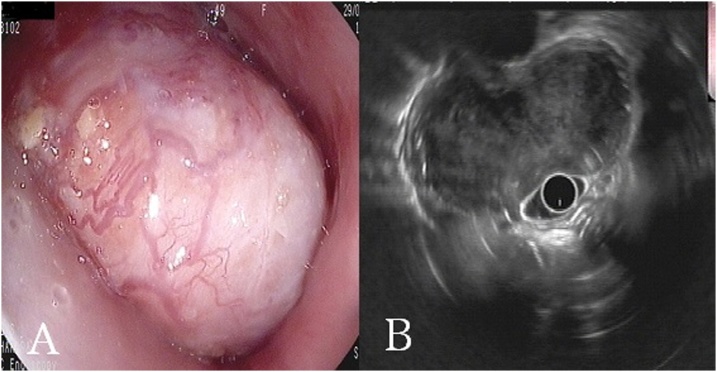
Upper gastrointestinal tract endoscopy revealing a protruding submucosal lesion at the proximal segment of the esophagus with an intact and hypervascular mucosa (**A**). The soft tissue mass is almost obstructing the esophageal lumen. (**B**) An endoscopic ultrasound image of the well-defined mass almost concealing the esophageal lumen.

An enhanced CT scan of the chest revealed a well-defined soft tissue mass arising from the proximal third of the esophagus, measuring 7.8*5.4*10.5 cm in its maximal transverse, AP, and CC dimensions, respectively, with prominent superior mediastinal lymph nodes, the largest measuring 7 mm (Fig. 1). An upper gastrointestinal endoscopy with ultrasound showed an elevated smooth surface lesion at around 20 cm from the incisors, originating from the submucosa, abutting the muscularis mucosa, and covered by a hypervascularized mucosa (Fig. 2).

Through a right posterolateral thoracotomy incision, the pathological portion of the esophagus was identified. The proximal portion of the esophagus was dilated due to the chronic partial obstruction caused by the mass.

**Fig. 3:**
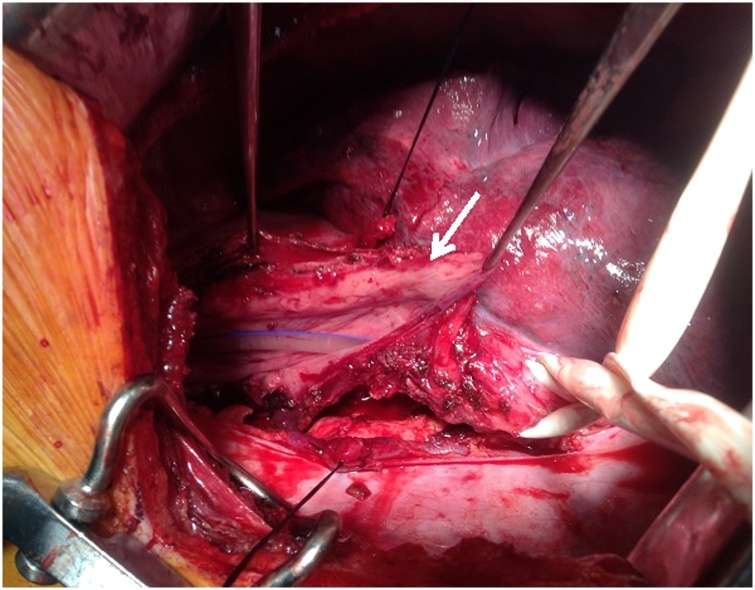
Intra-operative image showing the dilated residual proximal portion of the esophagus after complete resection of the schwannoma (*arrow*). Using this portion, a primary esophagoplasty closure was performed without compromising the esophageal caliber. Note the nasogastric tube within the lumen of the esophagus.

**Fig. 4:**
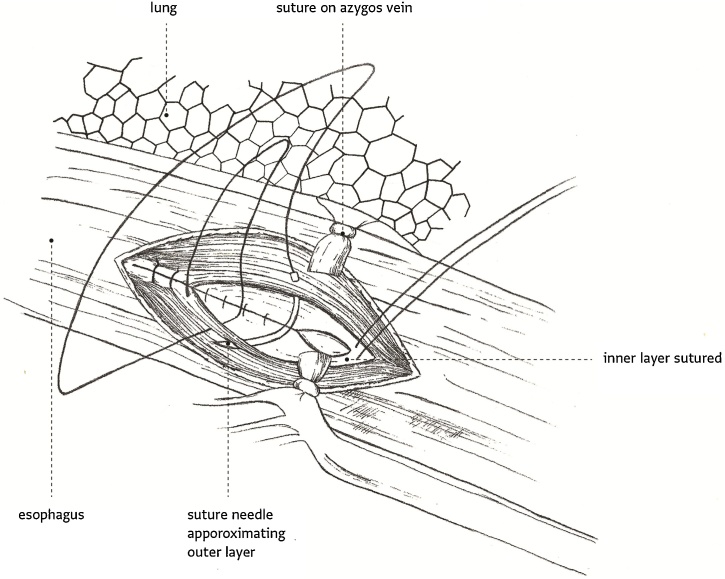
Illustrative drawing showing the longitudinal defect closure of the esophagus in two layers, using running 3-0 vicryl suture for the innermost mucosa and 3-0 Polydioxanone (PDS) for the muscular layer. The right lung, chest wall, and ligated azygous vein were shown for orientation purposes.

Enucleation of the mass was impossible as the layer between the mucosa and submucosa could not be developed safely. Taking advantage of a dilated esophageal segment proximally, en bloc excision of the tumor was performed preserving enough width of esophageal wall. The width of the remaining esophagus measured 8 cm uniformly along the 15 cm longitudinal defect caused by the tumor excision. The 8 cm width allowed the reconstruction of the 15 cm longitudinal defect of the esophagus without undue narrowing of the lumen (Fig. 3). The esophagus was closed in two layers using a 3-0 vicryl running suture for the mucosa and 3-0 Polydioxanone (PDS) for the muscular layers. (Fig. 4).

**Fig. 5:**
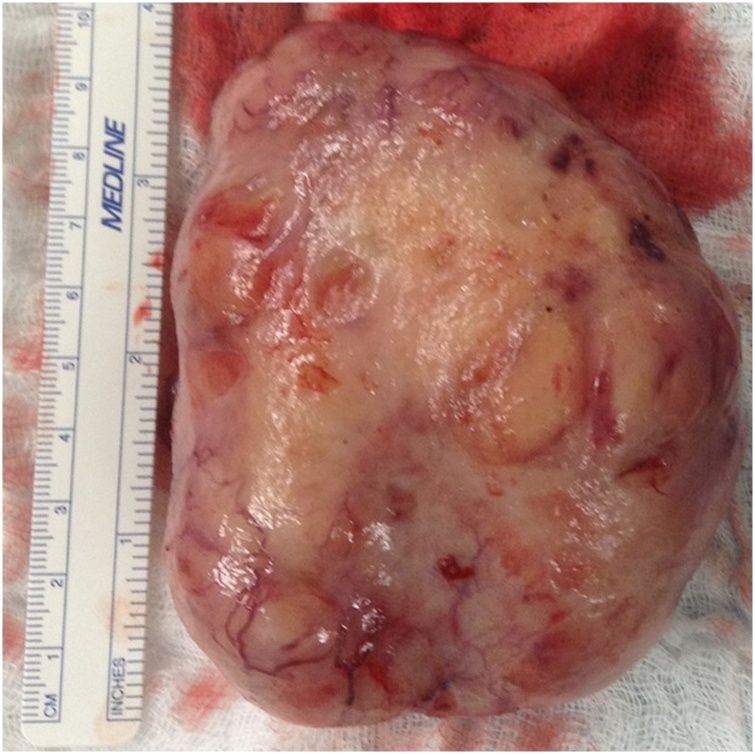
Gross specimen of the esophageal schwannoma after resection, measuring around 10 cm in longest dimension.

**Fig. 6:**
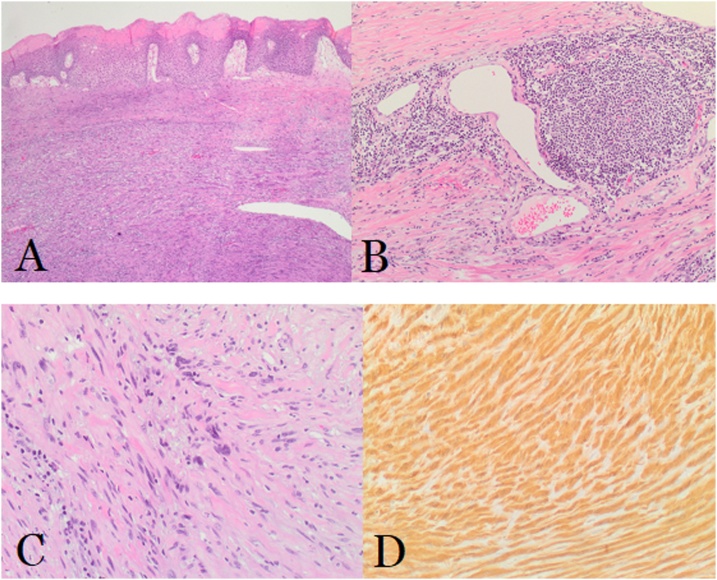
Histopathological examination using Hematoxylin and eosin stain of the specimen revealing bundles of spindle cells originating from the submucosal layer of the esophagus (**A; x40**) with the presence of lymphoid infiltrates and dilated angulated vessels at the edge of the tumor (**B; x100**). (**C; x200**) There is presence of ancient changes characterized by large atypical dark nuclei, which represent a peculiar component of schwannomas. (**D**) Immunostaining performed on formalin-fixed paraffin-embedded sections showing diffusely positive S-100 proteins. S-100 proteins are normally present in cells derived from the neural crest such as Schwann cells and others. Other immunostains including CD34, Desmin and CD117 were negative. Ki-67 labeling index was < 2%.

Grossly, a 9.5*7.0*3.0 cm firm mass, covered with normal mucosa having a focal ulceration, was identified (Fig. 5). The mass is tan-white, pale, and homogeneous, with neither necrotic nor hemorrhagic areas. Microscopically, the tumor was composed of compact bundles of spindle-shaped cells, arranged in a fasciculated and disarrayed architecture, and nuclei arranged in a palisading pattern (Fig. 6 A–C). Despite the presence of cytological atypia, no mitotic figures or necrosis were noted. Immunohistochemistry revealed a diffuse positivity for S-100 (Fig. 6D), focally positive SMA, and negative staining for CD34, CD117, and Desmin, respectively. This confirmed the diagnosis of a benign esophageal schwannoma. All lymph nodes in the resected specimen were negative.

Gastrograffin swallow, performed five days later, revealed no evidence of contrast leak. Diet resumed, gradually thereafter, and advanced as tolerated. She was discharged home after a smooth postoperative course. The patient has been followed up for approximately 4 years, without any evidence of recurrence nor complications.

## 3 Discussion

Esophageal schwannomas are categorized under neurogenic tumors. The latter are classified according to Ranson’s histopathological classification, published in 1940, as either nerve sheath tumors or neuroblastic tumors of the sympathetic system [7]. Schwannoma is the most common peripheral nerve sheath tumor. It usually occurs solitary and very rarely in the gastrointestinal tract [7].

Among the most common malignancies in the esophagus are the squamous cell carcinomas. Tumors that originate from mesenchymal cells, such as leiomyomas, leiomyosarcomas, gastrointestinal stromal tumors, and schwannomas, are uncommon inside the esophagus [8,9].

Schwannomas of the esophagus are frequently developed more in females than males, with a ratio of 4 to 1 [10], especially during the 5th to 6th decade of life. There is a strong predominance of this disease in Asian population, with most of reported cases from Asian institutions, in the world literature. The youngest reported case of esophageal schwannoma, by Choo et al was in a 22-year-old Asian American male, complaining of dyspnea with progressive dysphagia [11]. These tumors are frequently located in the upper to mid esophageal portion, within the mid mediastinum. So far, 98 cases of esophageal schwannomas are reported in the world literature (Appendix 1-Supplementary material).

Schwannoma of the gastrointestinal tract are submucosal tumors, commonly covered by normal mucosa and principally involving the submucosa and the muscularis propria [12]. Histologically, esophageal schwannoma portrays either Antoni A and/or B pattern(s) [1], and are characterized by peripheral lymphoid cuffing, benign nuclear atypia, and spindle-shaped cells [13]. Bundles of S-100 protein-positive spindle cells are intermixed in a fibrous, S-100 protein negative, background. They express negative expressions for smooth muscle markers such as actin, desmin, CD117(c-kit), and CD34. Hematoxylin and eosin staining reveals fascicular arrangement of spindle cells with palisading cell nuclei [5].

A preoperative including radiologically-alone diagnosis of this entity is difficult to achieve, and often established, after surgical resection [13].

As a rule, most benign mesenchymal tumors of the esophagus arise from the submucosa layer, and can be enucleated without destruction of the internal mucosa, and with preservation of good swallowing function. On the contrary, malignant neoplasms generally require more extensive resection and reconstruction; so the pre-emptive diagnosis is important in decision making, before any definitive treatment is undertaken [8].

Enucleation, using either video-assisted thoracoscopic surgery or endoscopically, is becoming the approach of choice for small submucosal tumors, measuring less than 2 cm in length [13,14]. Recently, robotic-assisted enucleation for a large esophageal schwannomas is made possible [15]. However, for large tumors, usually those greater than 8 cm with broad areas adherent to the muscularis layer, the resultant mucosal defect, upon resection, becomes extensive. In such scenarios, segmental esophagectomy, followed by esophagogastrostomy, are the preferred surgery of choice [16], as direct anastomosis is considered difficult. In general, any growing mass with symptoms are indications for surgical resection. As such, no cut off size of the tumor is mentioned in the literature. If it is noted as high grade on biopsy or more than 10 cm in size, the appropriate therapy is en-bloc esophagectomy with tumor-free resection margin, because those will carry malignant potentials [17].

Esophageal schwannomas can significantly increase in size, reaching up to 15 cm, as reported in the literature, without severe symptoms of dysphagia and dyspnea. In such extreme cases, there isn’t significant narrowing of the esophageal lumen or compression on the trachea. Variation in surgical approach is evident in the literature and depends mainly on the size and extent of the lesion. Simple lesion enucleation, segmental esophagectomy with primary anastomosis, or even subtotal esophagectomy with gastric pull-up or jejunal/colonic interposition are some of the surgical options. The originality in our report is that esophagoplasty as an option for repair of a large esophageal defect after en bloc resection of an esophageal schwannoma should be considered if the esophagus is dilated along the length of the tumor. The width of the remaining esophageal wall at the site of the tumor excision should be at least 7–8 cm to allow a longitudinal closure that should restore the normal caliber of the esophagus which is around 1.5–2.5 cm in diameter [18].

Malignant transformation, albeit rare in esophageal schwannomas, is indicated by the more frequent mitotic figures, necrosis, and cytological irregularities [1]. When malignancy is suspected, radical surgery is indicated, including esophagectomy and lymph node dissection, to avoid any recurrence or distant metastasis [1].

Finally, the prognosis with benign schwannomas of the gastrointestinal tract, after complete resection, is usually excellent [7], with dismal rate of recurrence.

## 4 Conclusion

Esophageal schwannomas are rare submucosal lesions that carry very low malignant potentials. While small lesions are amenable for enucleation using minimal invasive procedures, larger tumors, especially those greater than 8 cm, may request en-bloc esophagectomy. In cases where the proximal esophageal segment is dilated, resection followed by esophagoplasty for defect closure should be considered.

## Declaration of Competing Interest

The authors declare that they have no competing interests.

## Sources of funding

The authors declare any source of external funding for conducting this manuscript.

## Ethical Approval

The study such as this case report was exempted from ethical approval by the Institutional Review Board of the American University of Beirut-Medical Center.

## Consent

Written Informed consent was obtained from the patient for publication of this case report and any accompanying images. A copy of the written consent is available for review by the Editor-in-Chief of this journal.

Written informed consent was obtained from the patient for publication of this case report and accompanying images. A copy of the written consent is available for review by the Editor-in-Chief of this journal on request

## Authors’ Contributions

JD, PS, and AH performed the surgery for the patient. The manuscript was prepared by JD under the supervision of AH. Literature review was performed by JD, PS, and AH. IK performed the histological and immunological staining analysis of the gross specimen and provided images for the case report. All authors have approved the final version of the manuscript prior to submission.

## Registration of Research Studies

This is a case report not research study.

## Guarantor

Dr. Jad Degheili

## Provenance and peer review

Not commissioned, externally peer-reviewed
