# Supplementary material for: Large esophageal schwannoma: En-bloc resection with primary closure by esophagoplasty
Source: Int J Surg Case Rep. 2019 Jul 19;61:77–81. doi: 10.1016/j.ijscr.2019.07.038 (PMC6661383; doi:10.1016/j.ijscr.2019.07.038)
Supplement: Supplementary file 1 [file mmc1.docx]

**Table 1 (Supplementary material):**

| **No.** | **Reference (Year)** | **Age**  **(Years)/Sex** | **Chief Complaint** | **Size (cm)** | **Location** | **Operative method** | **Malignancy** | **Follow up**  **(months)** |
| --- | --- | --- | --- | --- | --- | --- | --- | --- |
| 1 | Chaterlin et al. (1967) |  |  |  |  |  |  |  |
| 2 | Fujieda et al. (1973) | 10/M | Swelling of left side of neck, dysphagia | 4.5*4.5*3.5 | CE | Enucleation | None | NA |
| 3 | Kato et al. (1982) | 37/F | Abnormal shadow on chest X-ray | 10.0*6.0*6.0 | LT | Enucleation | None | NA |
| 4 | Brooks et al. (1985) | 22/M | Pain on swallowing, dysphagia, 20Nonekg weight loss | 6.0 | CE | Incomplete excision | Yes | 23 |
| 5 | Madrid et al. (1986) | 53/F | Dysphagia, vomiting | 8.0*6.0*3.0 | CE | Polypectomy |  | NA |
| 6 | Koizumi et al. (1987) | 55/M | Unknown | 2.2*1.5*1.0 | AE | Endoscopic resection |  | NA |
| 7 | Konishi et al. (1989) | 79/M | Unknown | <0.5 | MT | Endoscopic resection | None | NA |
| 8 | Perch et al. (1991) | 22/M | Dysphagia, swelling in the neck | NA | CE | Surgical excision | Yes | 84 |
| 9 | Eberlein et al. (1992) | 62/M | Dysphagia, abdominal pain | 14.0*4.0*2.5 | CE | Polypectomy | None | 60 |
| 10 | Iwata et al. (1992) | 75/F | Cough, dyspnea | 10.0*8.0*5.0 | UT | Enucleation |  |  |
| 11 | Iwata et al. (1993) | 56/F | Abnormal shadow on chest X-ray | 4.8*4.2*3.0 | UT | Enucleation | Yes | 28 |
| 12 | Arai et al. (1994) | 56/F | Dysphagia, back pain | 8.0*3.5*5.0 | MT | Enucleation | None | NA |
| 13 | Arai et al. (1994) | 64/F | Dysphagia | 6.0*6.5*5.0 | NA | Enucleation | None | NA |
| 14 | Kataoka et al. (1994) | 56/F | Unknown | 4.8*4.2*3.0 | NA | Enucleation |  | NA |
| 15 | Kataoka et al. (1994) | 75/F | Unknown | 10.0*8.0*5.0 | UT | Enucleation |  | NA |
| 16 | Nishinaka et al. (1995) | 52/F | Dysphagia, oppressive feeling in the chest | 7.0*5.0*5.0 | MT | Enucleation | None | NA |
| 17 | Morita et al. (1996) | 57/F | Dysphagia | 4.0*3.5*2.7 | UT | Enucleation | Yes | NA |
| 18 | Ishida et al. (1997) | 66/F | Abnormal shadow on chest X-ray | 5.0*4.1*3.5 | LT | Enucleation | None | NA |
| 19 | Nakamura et al. (1998) | 59/M | Hemo-sputum | 5.0*3.5*2.5 | MT | Thoracoscopic resection | None | NA |
| 20 | Hamoir et al. (1998) | 18/F | Dysphagia, dyspnea, cough | 8.0 | CE | Enucleation | None | NA |
| 21 | Prévot et al. (1999) | 64/F | Dysphagia | 4.5 | CE | Enucleation | None | NA |
| 22 | Ohno et al. (2000) | 49/F | Dysphagia | 3.0*1.5*1.0 | UT | Enucleation | None | NA |
| 23 | Kobayashi et al. (2000) | 62/F | Asymptomatic | 3.4*2.8*2.6 | MT | Enucleation | None | 32 |
| 24 | Cokelaere et al. (2000) | 51/F | Dysphagia, odynophagia | 5.5*3.6*0.2 | CE | Enucleation | None | 10 |
| 25 | Manger et al. (2000) | 60/F | Dysphagia, swelling in the neck | 10.0*3.8*3.8 | LT | Partial Esophagectomy, esophagogastrostomy | Yes | 48 |
| 26 | Naus et al. (2001) | 39/M | Epigastric pain | 1.0*1.0*0.8 | LT | Endoscopic excision | None | 24 |
| 27 | Murase et al. (2001) | 49/F | Abnormal shadow on chest X-ray | 8.2*5.8*3.7 | MT | Enucleation | Yes | 16 |
| 28 | Kwon et al. (2002) | 70/F | Dysphagia, chest pain | 6.0 | MT | Surgical excision | None | 9 |
| 29 | Sato et al. (2002) | 55/M | Dysphagia | 8.5*7.0*4.0 | LT | Enucleation | Yes | NA |
| 30 | Lasota et al. (2003) | 36/F | NA | 3.5 | NA | Surgical excision | None | NA |
| 31 | Hsu et al. (2003) | 54/M | Dysphagia | 2.5*2.0*1.5 | LT | Enucleation | None | 16 |
| 32 | Hiranuma et al. (2003) | 64/F | Cough | 3.5*3.0*2.0 | MT | Enucleation (VATS) | None | NA |
| 33 | Tsuji et al. (2003) | 49/F | Dysphagia | 8.2*5.8*3.7 | MT | Enucleation | Yes | NA |
| 34 | Hoashi et al. (2003) | 58/F | None | 6.0*3.8*2.9 | MT | Esophagectomy | None | NA |
| 35 | Matsuhisa et al. (2004) | 66/M | None | 6.8*6.0*4.8 | LT-AE | Esophagectomy | None | NA |
| 36 | Horiuchi et al. (2004) | 72/M | Dysphagia | 5.5*4.5*4.5 | UT | Esophagectomy | None | NA |
| 37 | Sanchez et al. (2004) | 54/M | Dysphagia, weight loss | NA | AE | Ivor-Lewis procedure | Yes | 2 |
| 38 | Vinhais et al. (2004) | 48/F | Progressive dysphagia | 9.0 | MT | Enucleation | None | 36 |
| 39 | Saito et al. (2005) | 63/F | Dysphagia | 7.5*6.5*4.5 | UT | Enucleation | None | 10 |
| 40 | Sato et al. (2005) | 40/F | Dysphagia | 5.0*4.2*3.6 | UT | Enucleation | None | NA |
| 41 | Park et al. (2006) | 33/F | Dyspnea, right shoulder pain | 15.0*15.0*4.5 | LT | Total esophagectomy, esophago-gastric anastomosis | None | 12 |
| 42 | Basoglu et al. (2006) | 54/F | Dysphagia, weight loss, swelling in the left neck | 6.0*6.0 | CE | Total esophagectomy, esophago-gastric anastomosis | Yes | 40 |
| 43 | Vincent et al. (2006) | 54/F | Dysphagia, cough | 5.5 | CE | Segmental Cervical esophagectomy | None | 24 |
| 44 | Chen et al. (2006) | 73/F | Dysphagia, cough, dyspnea | 4.5*5.0*7.0 | UT | Enucleation (VATS) | None | 12 |
| 45 | Marin et al. (2006) | 54/F | Dysphagia, dry cough | 5.5 |  | Segmental resection of the esophagus and reconstruction | None | NA |
| 46 | Tokunaga et al. (2007) | 46/F | Dysphagia, dyspnea | 7.4*5.6*2.2 | UT | Enucleation | None | NA |
| 47 | Shigeoka et al. (2007) | 18/M | Unknown | Unknown | LT-MT | Esophagectomy | None | NA |
| 48 | Ito et al. (2007) | 78/F | Dysphagia | 8.5*4.3*4.6 | UT-MT | Esophagectomy | None | NA |
| 49 | Fukunaga et al. (2007) | 58/M | None | 5.0*3.5*3.0 | MT | Enucleation | None | NA |
| 50 | Ota et al. (2007) | 59/F | None | 2.1*1.2 | UT | Enucleation (VATS) | None | NA |
| 51 | Yoon et al. (2008) | 65/M | Dysphagia, swelling in the neck | 7.0*6.0*4.0 | CE | Enucleation | None | 24 |
| 52 | Mizuguchi et al. (2008) | 29/F | Severe dyspnea | 8.0*7.5*6.0 | UT | Enucleation (VATS) | None | 5 |
| 53 | Zhang et al. (2008) | 69/F | Left chest pain | 8.0*7.5*5.0 | UT | Enucleation | None | NA |
| 54 | Toyama et al. (2008) | 37/F | None | 2.8*2.4*1.9 | UT | Enucleation (VATS) | None | NA |
| 55 | Matsuki et al. (2009) | 73/F | Dysphagia, chest pain | 4.0*3.0*3.5 | UT | Enucleation | None | 14 |
| 56 | Mizuguchi et al. (2009) | 29/F | Dysphagia | 8.0*7.5*6.0 | UT | Enucleation (VATS) | None | NA |
| 57 | Fujita et al. (2009) | 41/M | Chest oppression | 4.5*4.3*3.4 | UT | Enucleation | None | NA |
| 58 | Kitami et al. (2009) | 62/M | Dysphagia | 3.7*5.6 | LT | Chemo-Radiation | Yes | NA |
| 59 | Dutta et al. (2009) | 52/F | Dysphagia, hematemesis | 6.0*5.0 | LT | Total esophagectomy, esophago-gastric anastomosis | None | NA |
| 60 | Retrosia et al. (2009) | 11/F | Neck mass, dysphagia, cough, dyspnea, pharyngodynia, dysphonia | 6.0 | UT | Enucleation | None | NA |
| 61 | Otaka et al. (2010) | 69/F | Dysphagia | 2.2*1.0*1.0 | CE | Enucleation | None | NA |
| 62 | Shien et al. (2010) | 66/F | Dysphagia | 5.0*4.5*3.5 | MT | Esophagectomy | None | NA |
| 63 | Shien et al. (2010) | 56/M | none | 2.5*2.0*1.7 | LT | Enucleation (VATS) | None | NA |
| 64 | Miyaki et al. (2011) | 75/M | Dysphagia | 4.5*3.2*4.0 | LT-AE | Esophagectomy | None | NA |
| 65 | Nakatsu et al. (2011) | 78/F | none | 5.0*3.0*3.0 | UT | Enucleation | None | NA |
| 66 | Nakatsu et al. (2011) | 70/F | Dysphagia | 4.0*3.5*2.7 | UT | Enucleation | None | NA |
| 67 | Makino et al. (2011) | 72/M | Asymptomatic | 2.2*3.4*2.9 | MT | Enucleation | None | 24 |
| 68 | Choo et al. (2011) | 22/M | Dysphagia, dyspnea, 20Nonekg weight loss | 8.5 | UT | Enucleation | None | 3 |
| 69 | Wang et al. (2011) | 44/F | Dysphagia | 5.5*4.0*4.5 | LT | Enucleation | Yes | 72 |
| 70 | Kassis et al. (2012) | 65/M | Paresthesia in the left hand | 11.3*8.4*5.8 | UT | Esophagectomy, cervical esophago-gastric anastomosis | None | 10 |
| 71 | Sato et al. (2012) | 43/F | none | 4.0*2.0 | UT | Enucleation (VATS) | None | NA |
| 72 | Liu et al. (2012) | 62/F | Dysphagia, dyspnea, | 9.0*4.0*3.0 | MT | Ivor-Lewis procedure | None | 3 |
| 73 | Kitada et al. (2013) | 55/F | Palpitations, discomfort during swallowing | 7.5*5.7*8.0 | MT | Sub-total esophagectomy, esophago-gastric anastomosis | None | NA |
| 74 | Dalci et al. (2014) | 61/F | Dysphagia, 10Nonekg weight loss | 8.0*6.0*4.0 | UT | Enucleation | None | 3 |
| 75 | Ferrante et al. (2014) | 69/F | Incidental finding post recurrent pneumonia and mild dysphagia | 5.0*2.3*2.0 | CE | Enucleation | None | NA |
| 76 | Ahn et al. (2014) | 36/F | Progressive Anterior neck mass; mild dysphagia | 6.5*4.5 | CE | Enucleation | None | NA |
| 77 | Jeon et al. (2014) | 63/M | Abnormal chest X-ray with widening mediastinum | 9.5*7.0*6.5 &  8.8*5.0*5.5 | UT | Enucleation | None | None |
| 78 | Jeon et al. (2014) | 32/F | Abnormal chest X-rays with widening mediastinum None intermittent chest pain | 8.7*5.9*2.4 | UT | Enucleation | None | None |
| 79 | Gu et al. (2014) | 39/M | Obstructive sensation during swallowing of several months duration | 3.5*3.2*1.2 | MT | Mass excision; VATS | None | NA |
| 80 | Kozak et al. (2015) | 37/F | Dysphagia | 3.5*3.0*3.0 | UT | Enucleation | None | NA |
| 81 | Wang et al. (2015) | 52/F | Neck discomfort | 3.0*2.5*2.2 | CE | Enucleation | None | NA |
| 82 | Wang et al. (2015) | 53/F | Neck mass | 3.0*2.0*2.0 | CE | Enucleation | None | NA |
| 83 | Tomono et al. (2015) | 59/F | Dysphagia, dyspnea, mass obstructing trachea | 10.9*7.2*7.1 | MT | Emergency subtotal esophagectomy | None | NA |
| 84 | Liu D et al (2015) | 62/F | Dysphagia of 1 month | ~ 9.0 cm | UT | Enucleation | None | NA |
| 85 | Shimamura Y. et al (2016) | 57/M | Acid-reflux symptoms | 0.5 cm | LT | Endoscopic mucosal resection | None | NA |
| 86 | Chen X et al (2016) | 46/M | Discomfort during swallowing | 3*2*1.7/  3*1.8*1.5 | UT | Enucleation | None | 12 |
| 87 | Chen X et al (2016) | 42/F | Dysphagia | 3*4*4 | UT | Enucleation | None | 48 |
| 88 | Chen X et al (2016) | 58/F | Dysphagia | 8*6*5 | UT | Enucleation | None | 60 |
| 89 | Mishra B et al (2016) | 27/F | Dysphagia; palpitations | 12*10*10 | UT | Esophagectomy; gastric conduit | Yes | 18 |
| 90 | Watanabe T et al (2016) | 39/F | Epigastric pain; dysphagia | 3.9*2.8*5.6 | UT | Thoracoscopic esophagectomy | None | NA |
| 91 | Hu Z et al (2017) | 57/F | Dysphagia; 2 years | 3.8*3.0 | UT | Thoracoscopic resection | None | NA |
| 92 | Onodera Yu et al (2017) | 47/F | Dysphagia | 6.0 | UT | Thoracoscopic resection | None | NA |
| 93 | Trindade A et al (2017) | 54/M | Reflux disease | 0.6 | LT | Endoscopic resection | None | NA |
| 94 | Wu C et al (2017) | 56/M | 4 months dysphagia | 3.7*5.0*2.3 | UT | Enucleation | None | 24 |
| 95 | Moro K et al (2017) | 66/M | Dysphagia | 5.2*4.0*3.1 | MT | Full thickness excision | None | 36 |
| 96 | Iwata Y et al (2018) | 74/F | Incidental finding | 8.0*4.2 | UT | Enucleation | None | 60 |
| 97 | An B et al (2018) | 74/F | Incidental finding after work up for lung cancer | 3.5*3.0 | LT | VATS | None | NA |
| 98 | Zhang Y. et al (2018) | 48/F | Dysphagia | 7.0 | LT-AE | Robotic-assisted enucleation | None | 50 |
| 99 | Present case (2019) | 50/F | Increasing onset of dyspnea and minimal dysphagia | 7.8x5.4x10.5 | UT | En-bloc resection, primary esophagoplasty | None | 48 |
|  |  |  |  |  |  |  |  |  |

F female, M male, NA not available, CE Cervical esophagus, UT upper thoracic esophagus, MT middle thoracic esophagus, LT lower thoracic esophagus, AE abdominal esophagus, VATS video-assisted thoracoscopy .
